# Identification and Characterization of a New Splicing Variant of Prokineticin 2

**DOI:** 10.3390/life12020248

**Published:** 2022-02-07

**Authors:** Roberta Lattanzi, Daniela Maftei, Martina Vincenzi, Maria Rosaria Fullone, Rossella Miele

**Affiliations:** 1 Department of Physiology and Pharmacology “Vittorio Erspamer”, Sapienza University of Rome, Piazzale Aldo Moro 5, 00185 Rome, Italy; daniela.maftei@uniroma1.it (D.M.); martina.vincenzi@uniroma1.it (M.V.); 2 Department of Biochemical Sciences “Alessandro Rossi Fanelli”, Sapienza University of Rome, Piazzale Aldo Moro 5, 00185 Rome, Italy; mariarosaria.fullone@uniroma1.it

**Keywords:** alternative splicing, prokineticin 2, prokineticin 2 splice variant, prokineticin receptors

## Abstract

Prokineticin 2 (PROK2) is a secreted bioactive peptide that regulates a variety of biological responses via two GPCRs, the prokineticin receptors (PROKRs). The aim of this study was to characterize a new alternatively spliced product of the *prok2* gene consisting of four exons. The 40-amino acid peptide, designated PROK2C, is encoded by exon 1 and exon 4, and its expression was detected in the hippocampus and spinal cord of mice. PROK2C was expressed in a heterologous system, *Pichia pastoris*, and its binding specificity to the amino-terminal regions of PROKR1 and PROKR2 was investigated by GST pull-down experiments. In addition, the introduction of the unnatural amino acid p-benzoyl-L-phenylalanine using amber codon suppression technology demonstrated the role of tryptophan at position 212 of PROKR2 for PROK2C binding by photoactivatable cross-linking. The functional significance of this new isoform was determined in vivo by nociceptive experiments, which showed that PROK2C elicits strong sensitization of peripheral nociceptors to painful stimuli. In order to analyze the induction of PROK2C signal transduction, STAT3 and ERK phosphorylation levels were determined in mammalian CHO cells expressing PROKR1 and PROKR2. Our data show by in vivo and in vitro experiments that PROK2C can bind and activate both prokineticin receptors.

## 1. Introduction

Alternative splicing (AS) is an important mechanism in gene modulation. It allows a single gene to produce several different mRNAs with different exon compositions and lengths that can code for multiple forms of the corresponding proteins.

Most eukaryotic protein-coding genes involved in developmental processes and regulation of cell proliferation have multiple transcriptional isoforms [1,2]. AS can affect mRNA localization, stability, and translation or change the reading frame, resulting in different protein isoforms with diverse functions, localizations, or both [3,4]. AS is highly regulated by several regulatory elements of splicing that modulate splice site selection and spliceosome assembly. Any alteration in the splicing mechanism can affect the maturation of mRNA and consequently the formation of functional proteins, causing various pathological states such as neurodegenerative diseases and inflammatory, immune, and metabolic disorders [5,6].

Prokineticin 2 (PROK2) is a chemokine-like protein found in all evolutionary scales, including reptiles, amphibians, mammals, and humans, and is characterized by finely regulated species-specific and cellular expression [7]. Structurally, PROK2 presents an AVITG N-terminal peptide sequence critical for its biological activity [8], ten identically spaced cysteine residues that define a five disulfide-bridged motif, and a Trp residue at position 24 that is critical for receptor binding [9].

In mammals, including humans, Chen and coworkers [10] have identified an alternative splice variant of the *prok2* gene named PROK2 long (PROK2L) which presents an insertion of 21 additional amino acids compared to PROK2, without any biological activity. Proteolytic cleavage of PROK2L gives rise to a smaller active form of the PROK2 protein called PROK2β.

PROK2 mRNA is detected in the testis, granulocytes, dendritic cells, macrophages, olfactory bulb, brain, lung, and spleen [7,11], whereas PROK2L mRNA expression is higher than PROK2 in the lung and spleen, and lower in the brain. Both PROK2 and PROK2L are present in adipocytes and the hypothalamus, where PROK2 and PROK2β regulate adipocyte differentiation and food intake [12,13].

PROK2 and PROK2β bind to two closely related G protein-coupled receptors (GPCRs), prokineticin receptor 1 and 2 (PROKR1 and PROKR2), with different selectivity: while PROK2 binds to both receptors with the same affinity, PROK2β binds preferentially to PROKR1 [10,14]. Depending on their cellular localization, both receptors can couple to a variety of G proteins and activate multiple intracellular signal transduction pathways [7]. Thus, receptor activation by both PROK2 and PROK2β has been shown to induce intracellular Ca^2+^ mobilization, stimulate cAMP accumulation, and activate ERK1/2, but only PROK2 induces STAT3 phosphorylation [12].

PROK2 and PROKRs have been shown to regulate numerous physiological and pathological processes, including angiogenesis, neurogenesis [15], circadian rhythms, hematopoiesis, feeding behavior, immune response, inflammation, and pain [7,16,17]. In rodents, PROK2 administration lowers pain thresholds to thermal and mechanical stimuli by activating PROKRs on nociceptors [18,19]. Furthermore, we have shown that PROK2β administration in rodents lowers pain threshold to thermal but not to mechanical stimuli by activating PROKR1 [14].

A new spliced variant of the *prok2* gene was recently identified and named PROK2C. The aim of this study was the molecular cloning and the pharmacological characterization of this alternatively spliced product.

## 2. Materials and Methods

### 2.1. PROK2C Production in Pichia pastoris

PROK2C was expressed as a His-tagged protein in *Pichia pastoris (P. pastoris*). The cDNA was obtained by RT-PCR using as template the total RNA with oligonucleotides reported in Table 1 (PROK2 up and PROK2 down) subcloned into PBS, digested with XhoI-NotI, and finally inserted in the *P. pastoris* integrative vector pPICzalpha fused to the α-factor sequence. This cloning strategy makes it possible to obtain a protein with the natural amino terminus and the carboxy terminal fused to the His-tag. Expression of PROK2C was performed as described in [9]. Crude culture supernatants were loaded onto 5 mL HiTrap^®^ IMAC FF in binding buffer 20 mM Tris HCl pH 7, 300 mM NaCl, 5 mM imidazole and eluted with 20 mM Tris-HCl (pH 7.0), 300 mM NaCl, 0.25 M imidazole. The recombinant ligand was pooled, dialyzed against 20 mM Tris-HCl (pH 7.0) buffer, and analyzed in Tris-Tricin SDS-PAGE gel and Western blot.

### 2.2. Animals

Three-month-old male wild-type (WT) C57BL/6J mice (Lexicon Genetics, The Woodlands, TX, USA), weighing 25–30 g, were used for the experiments. Four mice per cage were housed in a temperature-controlled environment (22 ± 2 °C) and maintained on a 12/12-h light/dark cycle with access to food and water ad libitum. All animal manipulations (drug administration, behavioral test, and sacrifice) were authorized by the Animal Care and Use Committee of the Italian Ministry of Health (number: 116/2015-PR) and performed in accordance with Directive 2010/63/EU of the European Parliament and Council of the European Union. All experiments involving animals were described in accordance with the ARRIVE guidelines 2.0 [20]. Every effort was made to minimize animal suffering and reduce the number of animals used.

### 2.3. Tissue Explants

Dorsal root ganglia (DRG), spinal cord, hippocampus, hypothalamus, adipose tissue, and myenteric plexus were collected from *n* = 4 WT mice, rapidly frozen on dry ice and stored at −80 °C for the subsequent RNA extraction.

### 2.4. RNA Extraction and qPCR

Total RNA was isolated from the DRG, spinal cord, hippocampus, hypothalamus, adipose tissue, and myenteric plexus using Trizol reagent (Thermo Fisher Scientific, Monza, Italy) as indicated by the manufacturer’s instructions. After spectrophotometric determination of concentration and purity, 1 μg of RNA was reverse transcribed into cDNA using the SensiFAST cDNA Synthesis Kit (Meridian Bioscience, Cincinnati, OH, USA), which was used as a template for qPCR. PCR amplification (iCycler; Bio-Rad, Milan, Italy) was performed on 50 ng of cDNA using DreamTaq DNA (Thermo Fisher Scientific, Monza, Italy) and specific mouse primers reported in Table 1 (PROK2C Fw and PROK2 Rev). All reactions were performed according to the same thermal protocol: 3 min at 95 °C for polymerase activation, 40 cycles at 95 °C for 30 s for denaturation phase, 30 s at 60 °C for annealing phase, and 1 min at 72 °C for extension phase. PCR amplification products were separated by electrophoresis on 2% agarose gel, visualized with gel red, and analyzed and photographed using the ChemiDoc XRS Imaging System (Bio-Rad, Milan, Italy).

### 2.5. Drug Administration

PROK2 and PROK2C were administrated by intraplantar route (i.pl.) in the right hind paw at the appropriate dilutions in a volume of 20 μL. Control animals received an equal volume of sterile saline solution (*n* = 8 mice per group).

### 2.6. Nociceptive Behavioral Test: Hot-Plate Test

Thermal hyperalgesia was assessed using the Hot-Plate Test. Mice were placed individually on a hot plate maintained at 48 °C and surrounded by a Plexiglas cylinder (Ugo Basile, Varese, Italy). The time (20 s of cut-off) of the first lick or flinch of the injected paw or jump to escape was recorded. Then, the animal was immediately removed. Basal sensitivity to thermal stimuli was measured before drug’s injection. The nociceptive threshold to the same thermal stimuli was measured before and at fixed time points after drug i.pl. administration. Groups of eight mice (*n* = 8) were used for each drug and dose.

The effect of the ligand tested was calculated as the percentage change in nociceptive threshold from baseline (%∆NT) according to the following equation: (%∆NT) = 100 × (NTTS − NTB)/NTB, where NTTS is the nociceptive threshold at that time point in the presence of the test solution and NTB is the baseline nociceptive threshold.

### 2.7. CHO-R1 and CHO-R2 Cell Culture and Stimulation

Chinese hamster ovary cells (CHO) stably expressing human PROKR1 or PROKR2 were cultured separately in Dulbecco’s Modified Eagle Medium/Nutrient mixture F-12 Ham (DMEM/F12; Sigma-Aldrich, Milan, Italy) supplemented with 10% fetal bovine serum (FBS, Sigma-Aldrich, Milan, Italy), 2 mM L-glutamine (Sigma-Aldrich, Milan, Italy), 100 U/mL penicillin/streptomycin (Sigma-Aldrich, Milan, Italy), and G418 200 ng/mL (Sigma-Aldrich, Milan, Italy) at 37 °C, 5% CO_2_. After serum starvation, cells were stimulated with PROK2 (100 nM) and PROK2C (100 nM) for 10 min and 1 h at 37 °C, 5% CO_2_. At the end of the incubation period, cells were lysed in protein extraction buffer, quantified by the Bradford method, and then used for Western blot analysis.

### 2.8. Analysis of STAT3 and ERK Activation in CHO-R1 and CHO-R2 by Western Blot Assay

Proteins were separated by electrophoresis and then transferred to a nitrocellulose membrane (TCM) and blocked in 1% nonfat milk 1% BSA/Tris-buffered saline containing 0.10% Tween-20 (TBS-T pH 7.4) for 1 h at room temperature. Subsequently, the membranes were incubated overnight at 4 °C with the appropriate primary antibodies rabbit anti-ERK (Santa Cruz, sc-153) and mouse anti-pERK (Cell Signaling Technology, Danvers, MA, USA, #9106S), mouse anti-STAT3 (MA1-13,042), and rabbit anti-pSTAT3(Tyr705) (# 44-380G) (Invitrogen-Thermo Fisher Scientific, Milan, Italy) diluted 1:1000 in the blocking solution. Membranes were then incubated with an anti-mouse IgG HRP-linked secondary antibody. The immunoreactive signals were visualized using an enhanced chemiluminescence system [12].

### 2.9. Glutathione S-Transferase (GST) Pull-Down

The R1-GST and R2-GST proteins were obtained by fusion of the 57 amino acids from the amino-terminal sequence of PROKR1 and the 47 amino acids from the amino-terminal sequence of PROKR2, respectively, with GST. R1- or R2-GST fusion proteins were purified from *Escherichia coli* cell extracts by affinity chromatography using glutathione-Sepharose beads (GE Healthcare, Little Chalfont, Buckinghamshire, UK) according to the manufacturer’s instructions. Briefly, 30 μL slurry of glutathione-Sepharose beads were equilibrated in buffer A (PBS, 1% Nonidet P 40, 1 mM EDTA supplemented with protease inhibitor mixture) for 1 h at 4 °C with constant stirring. The concentrations of GST fusion proteins, resuspended in buffer A, were determined by the BCA method (BCA Protein Analysis Reagents; Pierce, Thermo Fisher Scientific, Monza, Italy). Beads were incubated with an equivalent amount of purified PROK2 or PROK2C for 4 h, washed as described above, and bound proteins were eluted with GSH according to the procedure of GE Healthcare. Samples from the wash and elution fractions were analyzed on precast Bio-Rad Tricine 16% acrylamide gels according to the manufacturer’s protocol. Briefly, 10 µL of sample buffer (200 mM Tris-HCl, pH 6.8, 2% SDS, 40% glycerol, 0.04% Coomassie Brilliant Blue G-250, 2%100 mMβ-mercaptoethanol) was added, heated for 5 min at 90–95 °C, and electrophoresed for 100 min at 100 V at room temperature. The running buffers used were anode buffer 0.2 M Tris-Cl, pH 8.9, and cathode buffer 0.1 M Tris, 0.1 M Tricine, and 0.1% SDS. After electrophoresis, the gel was blotted and incubated with anti-His-labeled antibodies to visualize the protein bands.

### 2.10. Data Analysis

Data were analyzed using GraphPad Prism 6 for Windows by one-way ANOVA followed by Tukey post-test or two-way ANOVA followed by Bonferroni post-test when appropriate. Differences were considered significant at *p* < 0.05.

## 3. Results

### 3.1. Description of a New PROK2 Splicing Variant

The *prok2* gene consists of four exons and three introns and is characterized by evolutionarily highly conserved exon–intron junctions. Exon 1 encodes the signal peptide and the first five amino acids, the AVITG sequence, of the mature protein. Exon 2 encodes 42 amino acids, including 6 cysteines. Exon 3 encodes for a highly basic sequence of 21 amino acids. Exon 4 encodes for the remaining 34 amino acids, including the remaining 4 cysteines of the secreted protein.

**Figure 1:**
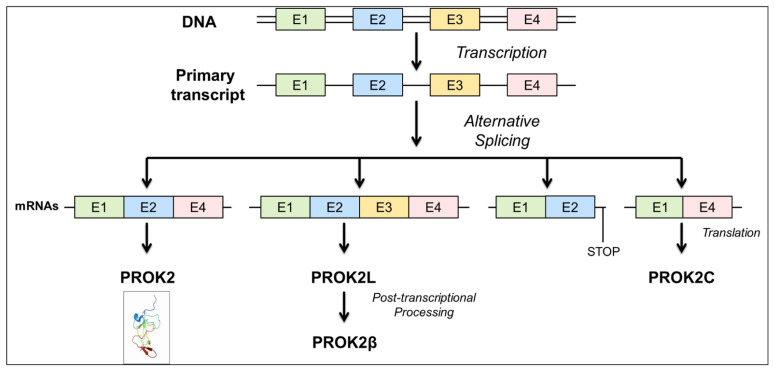
Schematic representation of *prok2* gene structure.

Computational analysis of the *prok2* gene (accession number: NP_001032628) identified four alternatively spliced mRNA transcripts in the mouse as shown in Figure 1.

The *prok2-203* transcript, consisting of exons 1, 2, and 4, encodes the canonical and most extensively characterized PROK2 isoform of 81 amino acids. The *prok2-202* transcript, consisting of exons 1, 2, 3, and 4, encodes the PROK2L isoform of 102 amino acids. PROK2L generates PROK2β by proteolytic cleavage. A third *prok2-204* transcript, consisting of exons 1 and 2 and part of intron 2, encodes a truncated PROK2 isoform of 86 amino acids that lacks both the basic portion and the COOH-terminal portion. The presence of this isoform has been demonstrated in the mouse brain [21]. The fourth *prok2-201* transcript, consisting of exons 1 and 4, encodes a 65-amino acid protein that we have named PROK2C.

### 3.2. PROK2C Expression in Mice

**Figure 2:**
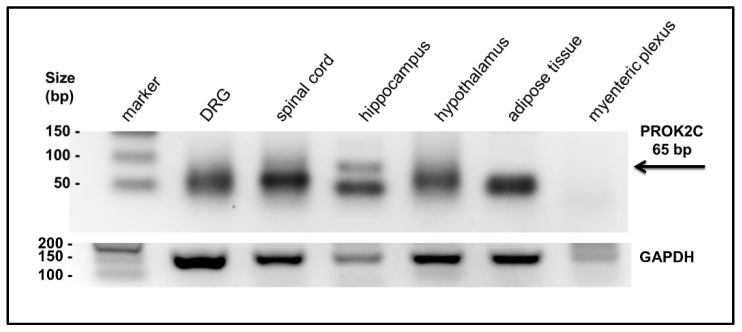
Expression of PROK2C in different mouse tissues. After qPCR, the PROK2C and GAPDH products were analyzed by agarose gel (2%) electrophoresis. A black band of the predicted PROK2C product was observed at 65 bp.

The presence of the PROK2C transcript was investigated by qPCR in different mouse tissues: DRG, spinal cord, hippocampus, hypothalamus, adipose tissue, and myenteric plexus. The amplified qPCR PROK2C products were obtained with an oligonucleotide that binds a region overlapping exon 1 and 4 and with an oligonucleotide corresponding to the C-terminal region of exon 4. By agarose gel electrophoresis, the presence of PROK2C was detected in the spinal cord and hippocampus (Figure 2).

### 3.3. Heterologous Expression and Purification of PROK2 in Pichia pastoris

PROK2C cDNA was amplified by PCR using PROK2 up and PROK2 dw oligonucleotides and mouse hippocampal cDNA as template. Sequence analysis of the three fragments obtained shows that one band encodes the full length of PROK2, one encodes PROK2L, and a third band corresponds to the mRNA encoding PROK2C. A cDNA of PROK2C was inserted into PICZ alfa and transformed into *P. pastoris*. Analysis of the yeast culture media by the SDS-PAGE shows that expression of the recombinant protein started from 48 h after methanol induction and the highest expression was observed in 96 h.

### 3.4. Analysis of the Interaction between PROK2C and the PROKR2 Receptor: Role of the Extracellular Loop 2

**Figure 3:**
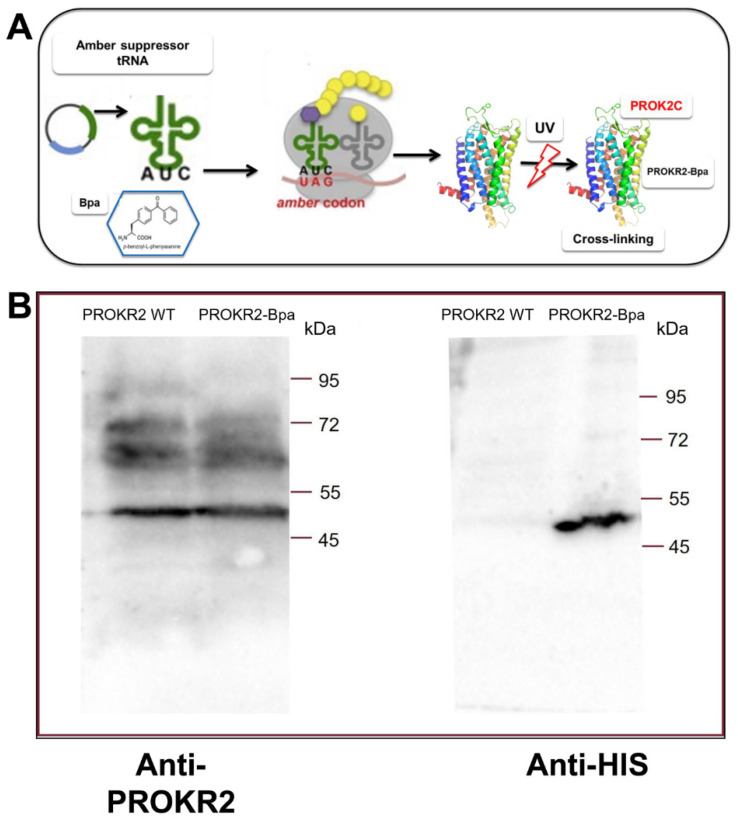
(**A**) Schematic representation of amber codon suppression technology for genetically introducing the photoreactive p-benzoyl-L-phenylalanine (Bpa) directly into expressed PKR2 in a yeast-cell-based system. (**B**) Cross-linking of PROK2C and PROKR2-Bpa receptor. Membranes (2 μg for WT and 25 μg for mutant) prepared from *P. pastoris* cells expressing the WT and W212 amber receptors, grown in the presence of Bpa, were incubated in presence of PROK2C (100 μM). Membrane proteins were immunoblotted and probed with anti-PROKR2 and anti-His antibodies.

In a yeast-cell-based system we utilized the codon suppression technology to genetically introduce the photoreactive p-benzoyl-L-phenylalanine (Bpa) at position 212 directly into expressed PKR2 (Figure 3). The plasmid encoding the amber PROKR2-W212 mutant was transformed in the *S. cerevisiae* strain Cy12946 with the plasmid pBpa2-PGK1+3SUP4-tRNACUA encoding the orthogonal amber suppressor tRNA synthetase/tRNA pair genetically modified to allow incorporation of Bpa [22], and with the plasmid that expresses the ligand PROK2. Whole-cell extracts of yeast strains were analyzed by Western blot using an antibody against PROKR2 and were shown to have similar expression levels of PROKR2 WT and the PROKR2-W212 amber mutant [23].

Membranes of *P. pastoris* cells expressing the amber receptors WT and PROKR2-W212 were incubated with or without PROK2C and then irradiated with UV light. The membranes were fractionated with SDS-PAGE, blotted, and then probed with the PROKR2 antibody (Figure 3A) to evidence the capture of ligands to the Bpa-labelled receptor. A distinct band of the size expected for PROKR2 was detected in the presence of PROKR2 WT such as PROKR2-W212Bpa. When the immunoblots were again reprobed with the PROK2 antibody, a distinct band of the size expected for PROK2C was detected only in the presence of PROKR2-W212Bpa (Figure 3B), indicating that the ligand forms a complex with PROKR2-W212Bpa by cross-linking. These results were confirmed by three independent replicates of this experiment.

### 3.5. Analysis of the Interaction of PROK2C with PROKR2 N-Terminal Region

**Figure 4:**
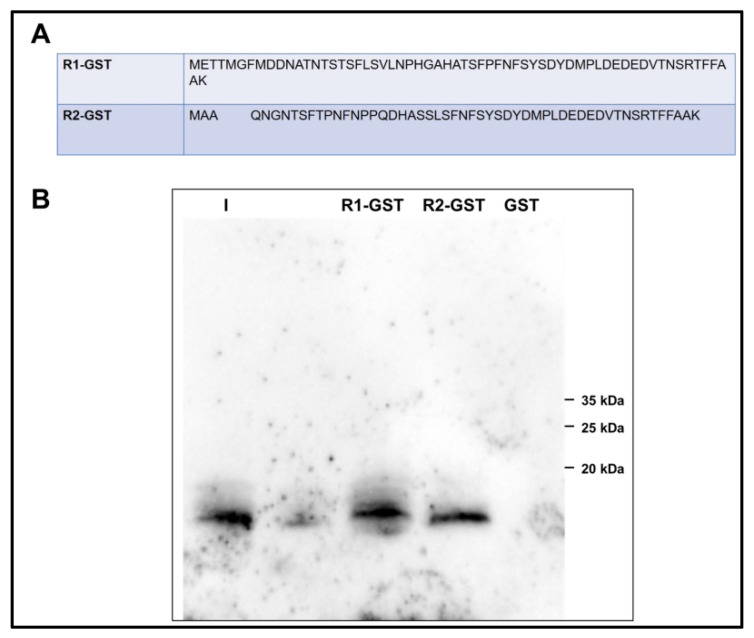
PROKR1 and PROKR2 amino-terminal regions bind PROK2C. (**A**) Alignment of the primary sequence of the amino-terminal region of human PROKR1 and PROKR2; (**B**) GST pull-down experiments. I, input; R1-GST, eluate; R2-GST, eluate; GST, glutathione S-transferase.

By glutathione S-transferase (GST) pull-down experiment, the interaction of the amino-terminal regions of PROKR1 and PROKR2 with ligands was analyzed. For this purpose, cell lysates from *E. coli* expressing GST protein fusions of the PROKR1 and PROKR2 amino-terminal regions (57 and 47 residues, respectively) were then plated onto glutathione-Sepharose columns and subsequently incubated with purified PROK2 or PROK2C. The amino-terminal regions of PROKR1 and PROKR2 were able to pull down PROK2C, suggesting that PROK2C is able to form a direct protein–protein interaction with PROKRs. Conversely, GST alone did not pull down PROK2C and PROK2 (Figure 4).

### 3.6. PROK2C Induces Thermal Hyperalgesia in Mice

It is known that, in WT mice, i.pl. administration of PROK2 at a dose of 65 fmol induces significant thermal hyperalgesia which peaks 30 min after administration and lasts for approximately two hours [14,18].

**Figure 5:**
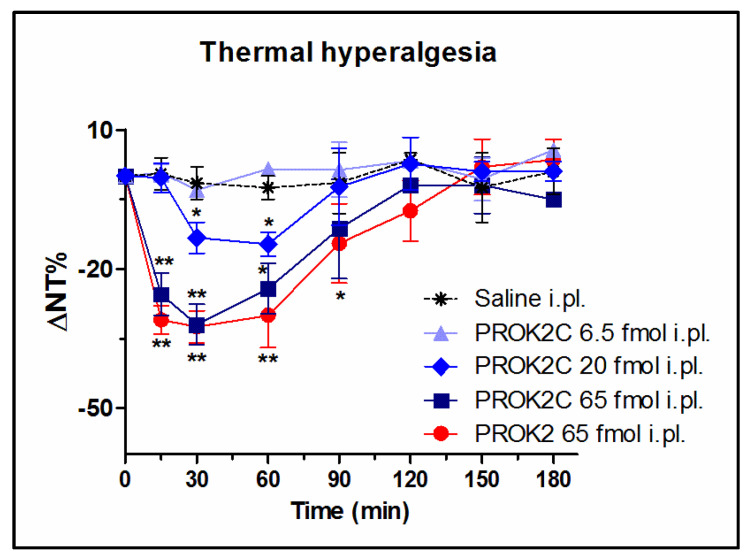
PROK2C induces thermal hyperalgesia in mice in a dose-dependent manner. Time-course of percent decrease in nociceptive threshold (% ΔNT) after i.pl. administration of PROK2 (65 fmol) and PROK2C (6.5, 20, and 65 fmol) induced by Hot Plate test at 48 °C in mice. Data represent means ± SEM of *n* = 8 mice. Two-way ANOVA followed by Bonferroni post-test was used for statistical evaluation ** *p* < 0.01, * *p* < 0.05 compared with saline group.

The ability of PROK2C to lower the nociceptive threshold to thermal stimuli was also investigated, using the Hot Plate test (48 °C), and compared with that induced by PROK2. Prior to drug administration the mice basal thermal nociceptive threshold was approximately 11.3 ± 0.5 s. PROK2C i.pl. injection at doses of 6.5, 20, and 65 fmol induced thermal hyperalgesia in a dose-dependent manner. In particular, the highest dose tested of 65 fmol induced a significant reduction of nociceptive threshold to heat noxious stimuli (31.5%, 7.7 ± 0.9 s), comparable to that induced by PROK2 (33%, 7.5 ± 0.6 s), with a peak 30 min after administration and a duration of approximately two hours. In contrast, the lower dose of 6.5 fmol had no effect on thermal hyperalgesia (Figure 5).

### 3.7. PROK2C Specifically Activates Prokineticin Receptor 1 and Prokineticin Receptor 2 in Mammalian Cells

**Figure 6:**
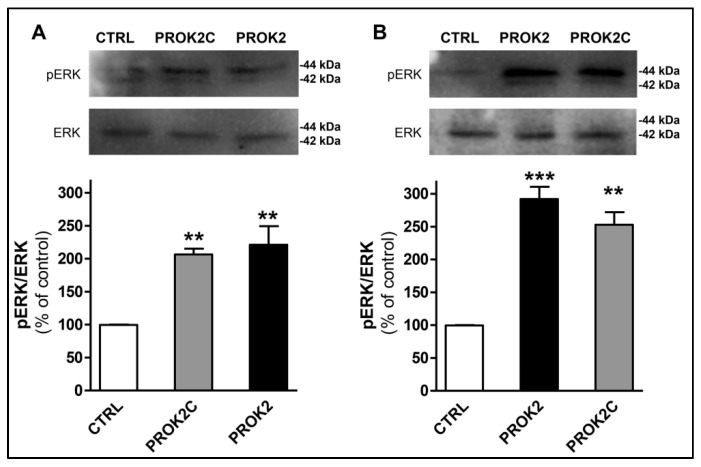
PROK2C induces ERK phosphorylation. Representative immunoblots and densitometric plots show phospho-ERK (pERK) and ERK protein levels in CHO cells expressing PROKR1 (**A**) or PROKR2 (**B**) receptors after 10 min of treatment with PROK2C (100 nM) and PROK2 (100 nM). Data are presented as a ratio of pERK to total ERK protein and plotted as % increase with respect to CTRL. Bar plots indicate means ± SEM obtained from the three experimental conditions. One-way ANOVA was used for statistical evaluation, followed by Tukey’s test for multiple comparisons ** *p* < 0.01, *** *p* < 0.001 vs. CTRL.

PROK2 has been shown to activate the ERK signaling pathway in endothelial cells [7] and in CHO cells expressing PROKR1 [24], and STAT3 via JAK2 in normal and malignant myeloid cells [25] and in DRG cells [14]. Therefore, we investigated whether PROK2C could also promote the activation of ERK and STAT3 in CHO cells stably expressing PROKR1 or PROKR2. The results showed that PROK2C induced significant ERK phosphorylation in both CHO cells expressing PROKR1 receptor and in those expressing PROKR2 receptor, 10 min after incubation (Figure 6). The observed effect was comparable to that induced by PROK2.

**Figure 7:**
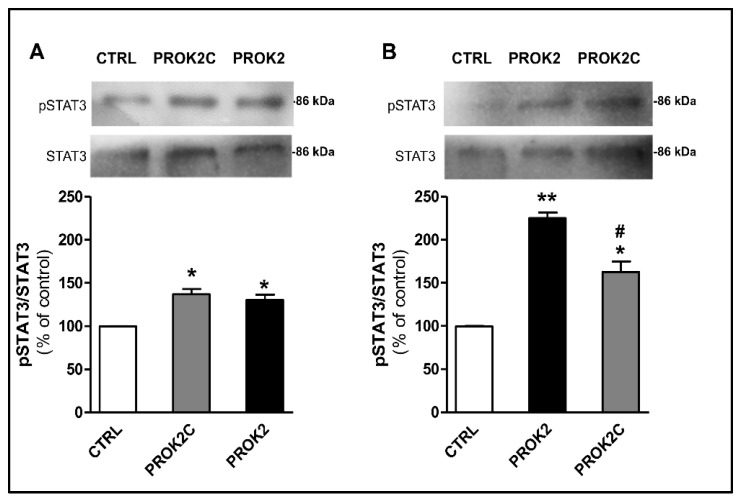
PROK2C induces STAT3 phosphorylation. Representative immunoblots and densitometric plots show phospho-STAT3 (pSTAT3) and STAT3 protein levels in CHO cells expressing PROKR1 (**A**) or PROKR2 (**B**) receptors after 1 h of treatment with PROK2C (100 nM) and PROK2 (100 nM). Data are presented as a ratio of pSTAT3 to total STAT3 protein and plotted as % increase with respect to CTRL. Bar plots indicate means ± SEM obtained from the three experimental conditions. One-way ANOVA was used for statistical evaluation, followed by Tukey’s test for multiple comparisons * *p* < 0.05, ** *p* < 0.01 vs. CTRL; # *p* < 0.05 vs. PROK2.

Similarly, incubation of CHO cells expressing PROKR1 or PROKR2 receptors with PROK2C induced a significant increase in phosphorylation of STAT3 at 1 h (Figure 7).

## 4. Discussion

The *prokineticin 2* gene and the *vegf* gene lead to different isoforms by alternative mRNA splicing, and their products both play a central role in angiogenesis and vasculogenesis [26].

PROK2C is encoded by exon 1 and exon 4. Exon 1, present in all PROK2 splice variants, encodes for the signal sequence that gives the protein the ability to be secreted and the AVITG sequence that is critical for receptor binding and induction of structural modification of the receptor. Exon 4 encodes the C-terminal PROK2 region. Unlike the other isoforms, PROK2C does not contain the region encoded by exon 2, including from alanine at position 6 to cysteine at position 41. This region contains important residues, both structurally (e.g., six cysteines) and functionally (e.g., a tryptophan at position 24 that has been shown to be essential for receptor binding) [9,27].

Recent structures of chemokine-bound receptors suggest the presence of two binding sites in the chemokine receptor: an extracellular site comprising the N-terminal region and ECL2, and a transmembrane orthosteric pocket [28].

The identification of these sites in PROKRs by computational analysis suggests a mechanism that, following ligand-specific interaction with the extracellular surface of the receptor, involves the insertion of AVITG into the orthosteric pocket. This induces a conformational modification of the receptor that allows intracellular signaling to be triggered [24,29].

The interaction of PROK2C with PROKRs’ TM-bundle binding site is ensured by the presence of the N-terminal region of AVITG.

To detect the interaction of PROK2C with the extracellular site of the receptor, we used two different approaches. We expressed in yeast a PROKR2 mutant that contained a substitution of Trp at position 212 with the unnatural amino acid Bpa. This mutant was used for cross-linking experiments showing that PROK2C could still interact with ECL2 despite the absence of Trp at position 24. GST pull-down technology was also used to demonstrate a specific interaction of PROK2C with the N-terminal region of PROKRs.

The ligand PROK2C was not only able to bind it, but also activated the prokineticin receptors. PROK2C showed an effect on pain behavior. Intraplantar injection of PROK2C produced antinociceptive sensitization to thermal stimuli comparable to that demonstrated for PROK2.

Similarly, after treatment of CHO cells stably expressing PROKR1 or PROKR2 with PROK2C, an increase in STAT3 and ERK phosphorylation was observed, which was comparable to the results obtained with PROK2.

## 5. Conclusions

The characterization of a new *prok2* gene splice variant, called PROK2C capable of binding and activating PROKRs receptors, encourages future studies on the role of this protein in the various signaling pathways involving the prokineticin system. Splicing is essential in normal physiological processes and its dysregulation can cause cellular dysfunction and disease. In light of this, future studies on PROK2 splicing mechanism is particularly promising as it can lead to the development of splicing modifying therapeutic drugs for the treatment of PROK2 triggered diseases.

## Figures and Tables

**Table 1:** Oligonucleotide sequences.

Oligonucleotide	Sequence	T (°C)
PROK2C-Fw	5′-CCGTGATCACCGGGGTTC-3′	66.0
PROK2C-Rv	5′-GAAGTCCGTAAACAGGCCAAG-3′	56.7
PROK2 up	5′-ATCTCGAGAAAAGAGCGGTCATCACCGGGGTTCCATTTTGGGGGCGG-3′	68
PROK2 dw	5′-TGGCGGCCGCTTTCCGGGCCAAGCAA-3′	64

## Data Availability

The data presented in this study are available on request from the corresponding authors.

## References

[1] Chen L., Bush S.J., Tovar-Corona J.M., Castillo-Morales A., Urrutia A.O. (2014). Correcting for differential transcript coverage reveals a strong relationship between alternative splicing and organism complexity. Mol. Biol. Evol..

[2] Graveley B.R. (2001). Alternative splicing: Increasing diversity in the proteomic world. Trends Genet..

[3] Chaudhary S., Khokhar W., Jabre I., Reddy A.S.N., Byrne L.J., Wilson C.M., Syed N.H. (2019). Alternative Splicing and Protein Diversity: Plants versus Animals. Front. Plant Sci..

[4] Kelemen O., Convertini P., Zhang Z., Wen Y., Shen M., Falaleeva M., Stamm S. (2013). Function of alternative splicing. Gene.

[5] Kim H.K., Pham M.H.C., Ko K.S., Rhee B.D., Han J. (2018). Alternative splicing isoforms in health and disease. Pflugers Arch..

[6] Sterne-Weiler T., Howard J., Mort M., Cooper D.N., Sanford J.R. (2011). Loss of exon identity is a common mechanism of human inherited disease. Genome Res..

[7] Negri L., Ferrara N. (2018). The Prokineticins: Neuromodulators and Mediators of Inflammation and Myeloid Cell-Dependent Angiogenesis. Physiol. Rev..

[8] Kaser A., Winklmayr M., Lepperdinger G., Kreil G. (2003). The AVIT protein family. Secreted cysteine-rich vertebrate proteins with diverse functions. EMBO Rep..

[9] Miele R., Lattanzi R., Bonaccorsi di Patti M.C., Paiardini A., Negri L., Barra D. (2010). Expression of Bv8 in Pichia pastoris to identify structural features for receptor binding. Protein Expr. Purif..

[10] Chen J., Kuei C., Sutton S., Wilson S., Yu J., Kamme F., Mazur C., Lovenberg T., Liu C. (2005). Identification and pharmacological characterization of prokineticin 2 beta as a selective ligand for prokineticin receptor 1. Mol. Pharmacol..

[11] Cheng M.Y., Leslie F.M., Zhou Q.Y. (2006). Expression of prokineticins and their receptors in the adult mouse brain. J. Comp. Neurol..

[12] Maftei D., Lattanzi R., Vincenzi M., Squillace S., Fullone M.R., Miele R. (2021). The balance of concentration between Prokineticin 2β and Prokineticin 2 modulates the food intake by STAT3 signaling. BBA Adv..

[13] Szatkowski C., Vallet J., Dormishian M., Messaddeq N., Valet P., Boulberdaa M., Metzger D., Chambon P., Nebigil C.G. (2013). Prokineticin receptor 1 as a novel suppressor of preadipocyte proliferation and differentiation to control obesity. PLoS ONE.

[14] Lattanzi R., Maftei D., Negri L., Fusco I., Miele R. (2018). PK2β ligand, a splice variant of prokineticin 2, is able to modulate and drive signaling through PKR1 receptor. Neuropeptides.

[15] Ng K.L., Li J.D., Cheng M.Y., Leslie F.M., Lee A.G., Zhou Q.Y. (2005). Dependence of olfactory bulb neurogenesis on prokineticin 2 signaling. Science.

[16] Giannini E., Lattanzi R., Nicotra A., Campese A.F., Grazioli P., Screpanti I., Balboni G., Salvadori S., Sacerdote P., Negri L. (2009). The chemokine Bv8/prokineticin 2 is up-regulated in inflammatory granulocytes and modulates inflammatory pain. Proc. Natl. Acad. Sci. USA.

[17] Negri L., Maftei D. (2018). Targeting the Prokineticin System to Control Chronic Pain and Inflammation. Curr. Med. Chem..

[18] Maftei D., Vellani V., Artico M., Giacomoni C., Severini C., Lattanzi R. (2020). Abnormal Pain Sensation in Mice Lacking the Prokineticin Receptor PKR2: Interaction of PKR2 with Transient Receptor Potential TRPV1 and TRPA1. Neuroscience.

[19] Negri L., Lattanzi R., Giannini E., Colucci M., Margheriti F., Melchiorri P., Vellani V., Tian H., De Felice M., Porreca F. (2006). Impaired nociception and inflammatory pain sensation in mice lacking the prokineticin receptor PKR1: Focus on interaction between PKR1 and the capsaicin receptor TRPV1 in pain behavior. J. Neurosci..

[20] Percie du Sert N., Ahluwalia A., Alam S., Avey M.T., Baker M., Browne W.J., Clark A., Cuthill I.C., Dirnagl U., Emerson M. (2020). Reporting animal research: Explanation and elaboration for the ARRIVE guidelines 2.0. PLoS Biol..

[21] Jilek A., Engel E., Beier D., Lepperdinger G. (2000). Murine Bv8 gene maps near a synteny breakpoint of mouse chromosome 6 and human 3p21. Gene.

[22] Chen S., Schultz P.G., Brock A. (2007). An improved system for the generation and analysis of mutant proteins containing unnatural amino acids in Saccharomyces cerevisiae. J. Mol. Biol..

[23] Fullone M.R., Lattanzi R., Maftei D., Bonaccorsi M.C., Miele R. (2021). Analysis of role of aromatic residues in extracellular loop 2 of Prokineticin receptor 2 in ligand binding probed with genetically encoded photo-crosslinkers. Biochim. Biophys. Acta Biomembr..

[24] Gasser A., Brogi S., Urayama K., Nishi T., Kurose H., Tafi A., Ribeiro N., Désaubry L., Nebigil C.G. (2015). Discovery and cardioprotective effects of the first non-Peptide agonists of the G protein-coupled prokineticin receptor-1. PLoS ONE.

[25] Xin H., Lu R., Lee H., Zhang W., Zhang C., Deng J., Liu Y., Shen S., Wagner K.U., Forman S. (2013). G-protein-coupled receptor agonist BV8/prokineticin-2 and STAT3 protein form a feed-forward loop in both normal and malignant myeloid cells. J. Biol. Chem..

[26] Harper S.J., Bates D.O. (2008). VEGF-A splicing: The key to anti-angiogenic therapeutics?. Nat. Rev. Cancer.

[27] Negri L., Lattanzi R. (2012). Bv8/PK2 and prokineticin receptors: A druggable pronociceptive system. Curr. Opin. Pharmacol..

[28] Kufareva I., Salanga C.L., Handel T.M. (2015). Chemokine and chemokine receptor structure and interactions: Implications for therapeutic strategies. Immunol. Cell. Biol..

[29] Levit A., Yarnitzky T., Wiener A., Meidan R., Niv M.Y. (2011). Modeling of human prokineticin receptors: Interactions with novel small-molecule binders and potential off-target drugs. PLoS ONE.

